# Ciprofloxacin exacerbates dysfunction of smooth muscle cells in a microphysiological model of thoracic aortic aneurysm

**DOI:** 10.1172/jci.insight.161729

**Published:** 2023-01-24

**Authors:** Bitao Xiang, Mieradilijiang Abudupataer, Gang Liu, Xiaonan Zhou, Dingqian Liu, Shichao Zhu, Yang Ming, Xiujie Yin, Shiqiang Yan, Yongxin Sun, Hao Lai, Chunsheng Wang, Jun Li, Kai Zhu

**Affiliations:** 1 Department of Cardiac Surgery, and; 2 Shanghai Institute of Cardiovascular Diseases, Zhongshan Hospital, Fudan University, Shanghai, China.; 3 Institutes of Biomedical Sciences and Shanghai Key Laboratory of Medical Epigenetics, Shanghai Medical College, and; 4 The State Key Laboratory of Molecular Engineering of Polymers, Fudan University, Shanghai, China.

**Keywords:** Vascular Biology, Apoptosis, Cardiovascular disease, Drug screens

## Abstract

Ciprofloxacin use may be associated with adverse aortic events. However, the mechanism underlying the effect of ciprofloxacin on the progression of thoracic aortic aneurysm (TAA) is not well understood. Using an in vitro microphysiological model, we treated human aortic smooth muscle cells (HASMCs) derived from patients with bicuspid aortic valve– or tricuspid aortic valve–associated (BAV- or TAV-associated) TAAs with ciprofloxacin. TAA C57BL/6 mouse models were utilized to verify the effects of ciprofloxacin exposure. In the microphysiological model, real-time PCR, Western blotting, and RNA sequencing showed that ciprofloxacin exposure was associated with a downregulated contractile phenotype, an upregulated inflammatory reaction, and extracellular matrix (ECM) degradation in the normal HASMCs derived from the nondiseased aorta. Ciprofloxacin induced mitochondrial dysfunction in the HASMCs and further increased apoptosis by activating the ERK1/2 and P38 mitogen–activated protein kinase pathways. These adverse effects appeared to be more severe in the HASMCs derived from BAV- and TAV-associated TAAs than in the normal HASMCs when the ciprofloxacin concentration exceeded 100 μg/mL. In the aortic walls of the TAA-induced mice, ECM degradation and apoptosis were aggravated after ciprofloxacin exposure. Therefore, ciprofloxacin should be used with caution in patients with BAV- or TAV-associated TAAs.

## Graphical abstract

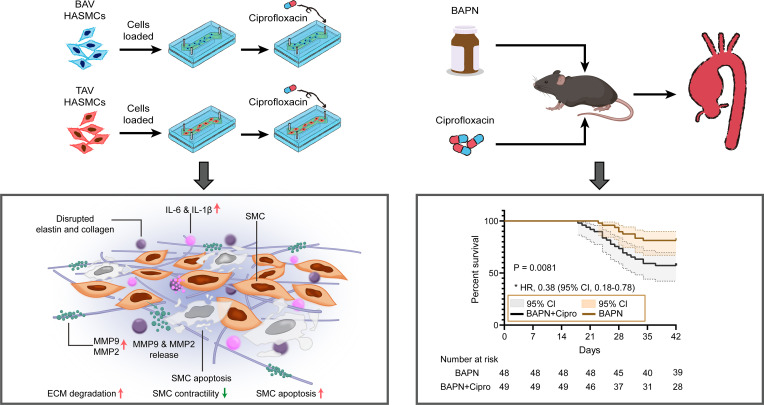

## Introduction

Thoracic aortic aneurysm (TAA) is a multifactorial cardiovascular disease with a substantial risk for aortic dissection (AD) or rupture. Certain factors are known to influence the development of TAA, including aging, sex, connective tissue disorders, atherosclerosis, smoking, hypertension, and family history (1, 2). Fluoroquinolones are among the most prescribed classes of antibiotics, owing to their broad-spectrum coverage, excellent oral bioavailability, extensive tissue penetration, and historically few adverse effects (3, 4). Recently, researchers found that fluoroquinolone use poses an increased risk of developing aortic aneurysm (AA)/AD. Furthermore, the risk of adverse outcomes from fluoroquinolone exposure is high in patients with AA/AD (5–13). Previous studies raise concerns regarding the use of fluoroquinolones in high-risk populations. However, it is difficult to investigate the underlying mechanism of fluoroquinolones in patients with TAA by conducting clinical trials, which could be potentially harmful and lethal under drug exposure. Therefore, TAA animal models, including Marfan syndrome–associated and sporadic TAA models, have been used in recent studies to investigate fluoroquinolone exposure (12, 13). However, drug responses in animal models cannot reflect the real mechanisms in humans because of the massive species gap. Moreover, the currently used TAA animal models merely represent partial types of TAAs. For instance, bicuspid aortic valve–associated (BAV-associated) TAAs have not been explored under ciprofloxacin exposure because it is difficult to construct animal models with effective BAV-associated TAA penetrance (14). The organ-on-a-chip model is a system containing engineered or natural miniature tissues grown inside microfluidic chips. Although this model cannot simulate the entire structure and complete function of an organ, it can simulate specific functional units by culturing living cells in micrometer-sized chambers with different stimuli to replicate the key functions of a tissue or organ. To date, several organ-on-a-chip models, such as those for the lung (15), kidney (16), liver (17), intestine (18), and heart (19), have been established to simulate specific functional units. In our previous study, we first established a human aortic smooth muscle–on-a-chip model that simulates the cyclic strain experienced by human aortic smooth muscle cells (HASMCs), which plays a critical role in aortic diseases (20). Under cyclic strain conditions, HASMCs present a higher percentage of the contractile phenotype and align unidirectionally, which approximates their characteristics in the native human aortic wall (20), thus providing an excellent in vitro model for the study of aortopathy and drug testing. In this study, we engineered patient-derived microphysiological models using HASMCs derived from patients with BAV- or tricuspid aortic valve–associated (TAV-associated) TAAs and treated them with ciprofloxacin, one of the most widely used fluoroquinolones. Our previous study has shown that apoptosis of HASMCs is associated with impaired mitochondrial dynamics (20). In addition, ciprofloxacin can augment the apoptotic effects of cisplatin in human pancreatic cancer cells by activating the mitogen-activated protein kinase (MAPK) signaling pathway (21), which is one of the critical regulatory pathways of cell apoptosis (22). Thus, we hypothesized that ciprofloxacin may induce mitochondrial dysfunction and further lead to HASMC apoptosis in patients with BAV- and TAV-associated TAAs by activating the MAPK signaling pathway.

## Results

### Fabrication of a patient-derived aortic microphysiological model.

**Figure 1:**
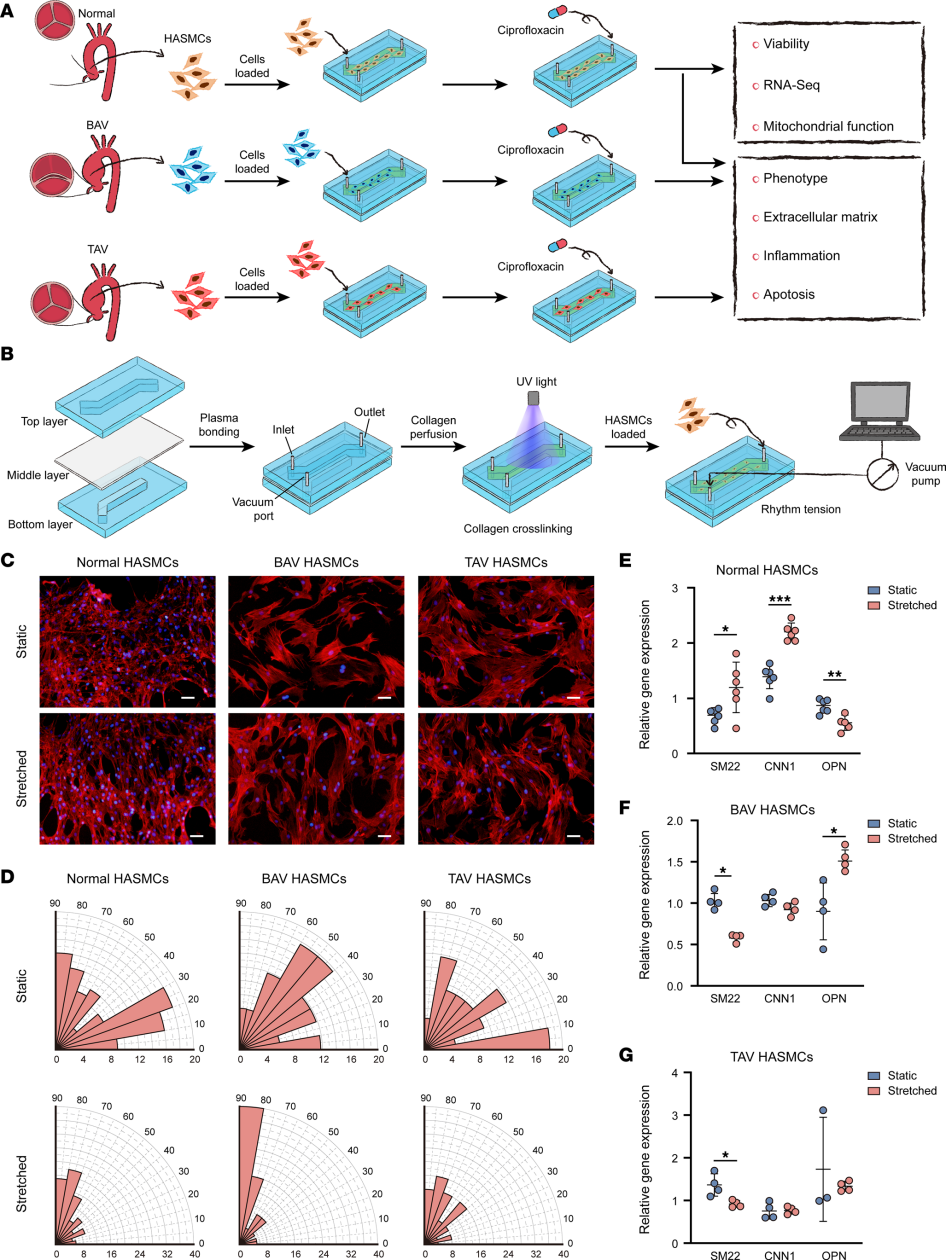
Construction of the human aortic microphysiological model. (**A**) Schematic workflow of the experimental process. (**B**) Schematic workflow of the fabrication process of the microphysiological model. (**C**) Immunofluorescent staining with rhodamine phalloidin of the normal HASMCs and HASMCs derived from patients with BAV- and TAV-associated TAAs. Scale bars: 100 μm. (**D**) Alignments of the normal HASMCs and HASMCs derived from patients with BAV- and TAV-associated TAAs. Relative mRNA levels of SM22, CNN1, and OPN measured by RT-PCR and normalized to β-actin as an internal control from (**E**) the normal HASMCs (*n* = 6), (**F**) HASMCs derived from patients with BAV-associated TAAs (*n* = 4), and (**G**) HASMCs derived from patients with TAV-associated TAAs (*n* = 4). *P < 0.05, **P < 0.01, ***P < 0.001 by 2-tailed Student’s *t* test.

Using a microphysiological model, we tested the biological responses of HASMCs to ciprofloxacin treatment from different aspects (Figure 1A). A schematic overview of the fabrication process for the microphysiological model is shown in Figure 1B. Rhodamine phalloidin–stained images of the HASMCs indicated that rhythmic strain increased the cell length and caused the cells to align perpendicular to the direction of the applied strain (Figure 1, C and D). Under rhythmic strain conditions, the mRNA expression of smooth muscle 22 (SM22) (*P* = 0.03) and calponin 1 (CNN1) (*P* < 0.0001), which are markers of the contractile phenotype in HASMCs, was significantly upregulated; meanwhile, the expression of the synthetic phenotype gene encoding osteopontin (OPN) (*P* < 0.001) was significantly downregulated under the normal condition compared with that under the static condition (Figure 1E). In the BAV-HASMC group, the SM22 mRNA expression was downregulated (*P* = 0.03), while the OPN mRNA expression was upregulated (*P* = 0.02) under rhythmic strain conditions (Figure 1F). Similarly, the SM22 mRNA expression was downregulated in the TAV-HASMC group under rhythmic strain conditions (*P* = 0.02, Figure 1G). These results suggest that the microphysiological model can replicate the physiological and pathological properties of HASMCs in the aortic wall.

### Effects of ciprofloxacin on the contractile phenotype, extracellular matrix, and proinflammatory properties of the HASMCs.

**Figure 2:**
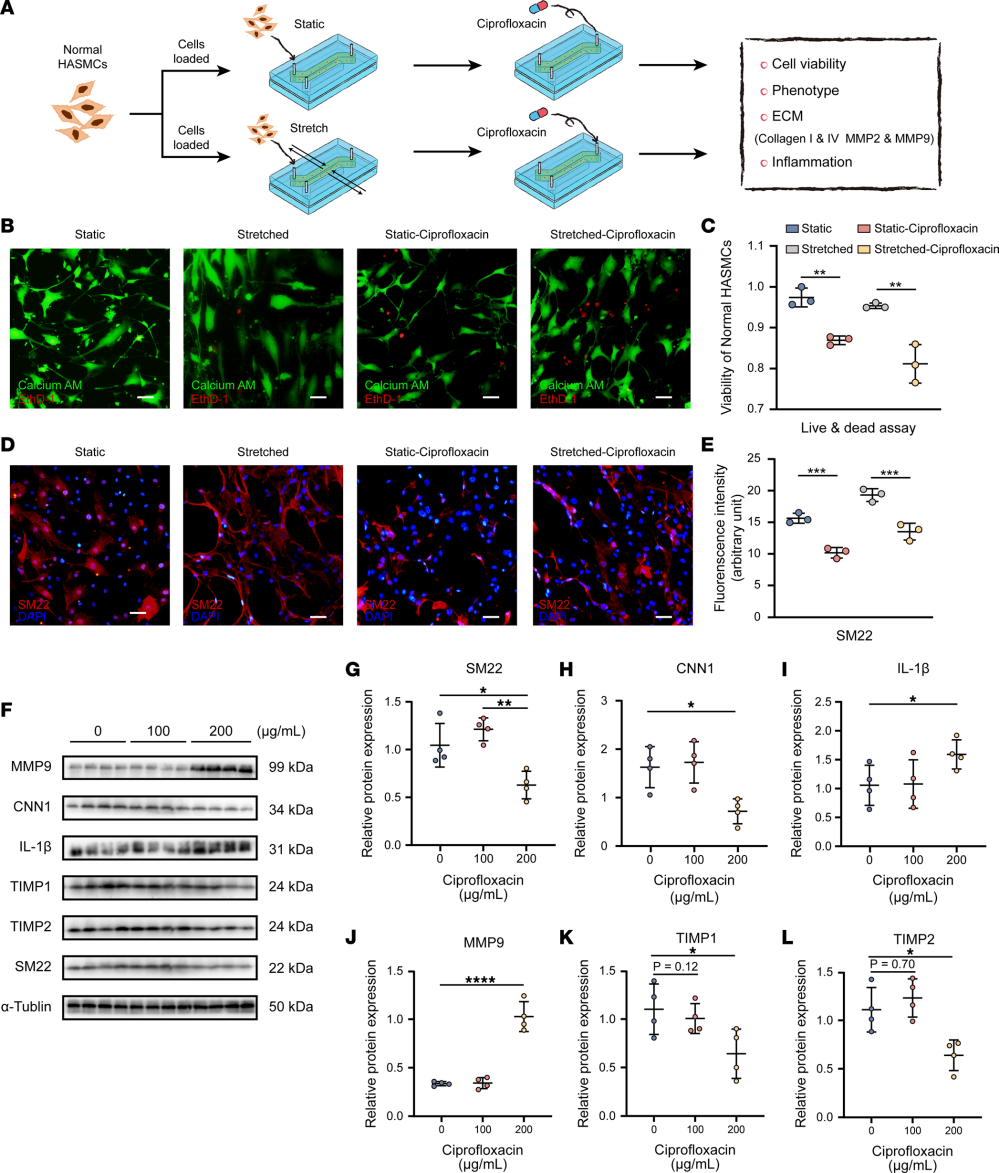
Ciprofloxacin induced ECM degradation and upregulated inflammation in the normal HASMCs. (**A**) Schematic workflow of testing the impact of ciprofloxacin on the normal HASMCs under static or strain condition in the microphysiological model. (**B**) Live (calcium AM) and dead (EthD-1) cells were stained after ciprofloxacin treatment (200 μg/mL) under static or strain condition (*n* = 3). Scale bars: 100 μm. (**C**) The viability of the HASMCs reduced after ciprofloxacin treatment (200 μg/mL) under static and strain conditions. (**D** and **E**) The immunofluorescent staining of SM22 decreased after ciprofloxacin treatment (200 μg/mL) under static and strain conditions (*n* = 3). Scale bars: 100 μm. (**F**–**L**) Representative images of Western blotting of SM22, TIMP1, TIMP2, IL-1β, CNN1, and MMP9 and quantification of the total band densities normalized to the corresponding band density of α-tubulin (*n* = 4). *P < 0.05; **P < 0.01; ***P < 0.001; ****P < 0.0001 by 1-way ANOVA with Tukey’s multiple-comparison test.

The effect of ciprofloxacin on the viability, phenotype, extracellular matrix (ECM), and proinflammatory properties of the HASMCs was investigated; the schematic workflow chart is shown in Figure 2A. A cell counting kit-8 (CCK-8) assay was performed to evaluate the viability of the HASMCs, which showed that ciprofloxacin decreased the viability of the normal HASMCs in a dose-dependent manner (Supplemental Figure 1; supplemental material available online with this article; https://doi.org/10.1172/jci.insight.161729DS1). A 200 μg/mL concentration of ciprofloxacin was used in the experiments on the models. To compare the low-dose drug response between different groups, we also used a 100 μg/mL concentration of ciprofloxacin. Subsequently, we performed a live/dead test to assess the influence of ciprofloxacin on the viability of the HASMCs (Figure 2B), which further demonstrated the adverse effects of ciprofloxacin on cell viability (Figure 2C). Immunofluorescent staining of markers of the contractile phenotype showed that the SM22 expression was downregulated in the ciprofloxacin-treated HASMCs under both static and rhythmic strain conditions compared with that in the non–ciprofloxacin-treated cells (Figure 2, D and E).

The mRNA expression of collagen (COL) I and contractile phenotype genes encoding SM22 and CNN1 (Supplemental Figure 2, A and B) was downregulated, while that of proinflammatory factors IL-6 and IL-1β (Supplemental Figure 2C) and matrix metalloproteinase 2 and 9 (MMP2 and -9) (Supplemental Figure 2D) was upregulated in the 200 μg/mL ciprofloxacin treatment group. Similar results were verified at the protein level by Western blotting (Figure 2, F–J). Moreover, the expression of the tissue inhibitors of matrix metalloproteinase 1 and (TIMP1 and -2) was significantly downregulated at 200 μg/mL (*P* < 0.05, Figure 2, K and L). These results indicate that ciprofloxacin has an adverse effect on HASMCs, presumably by downregulating the contractile phenotype and aggravating the inflammatory reaction and ECM degradation.

### Effects of ciprofloxacin on apoptosis and mitochondrial dysfunction in the HASMCs.

**Figure 3:**
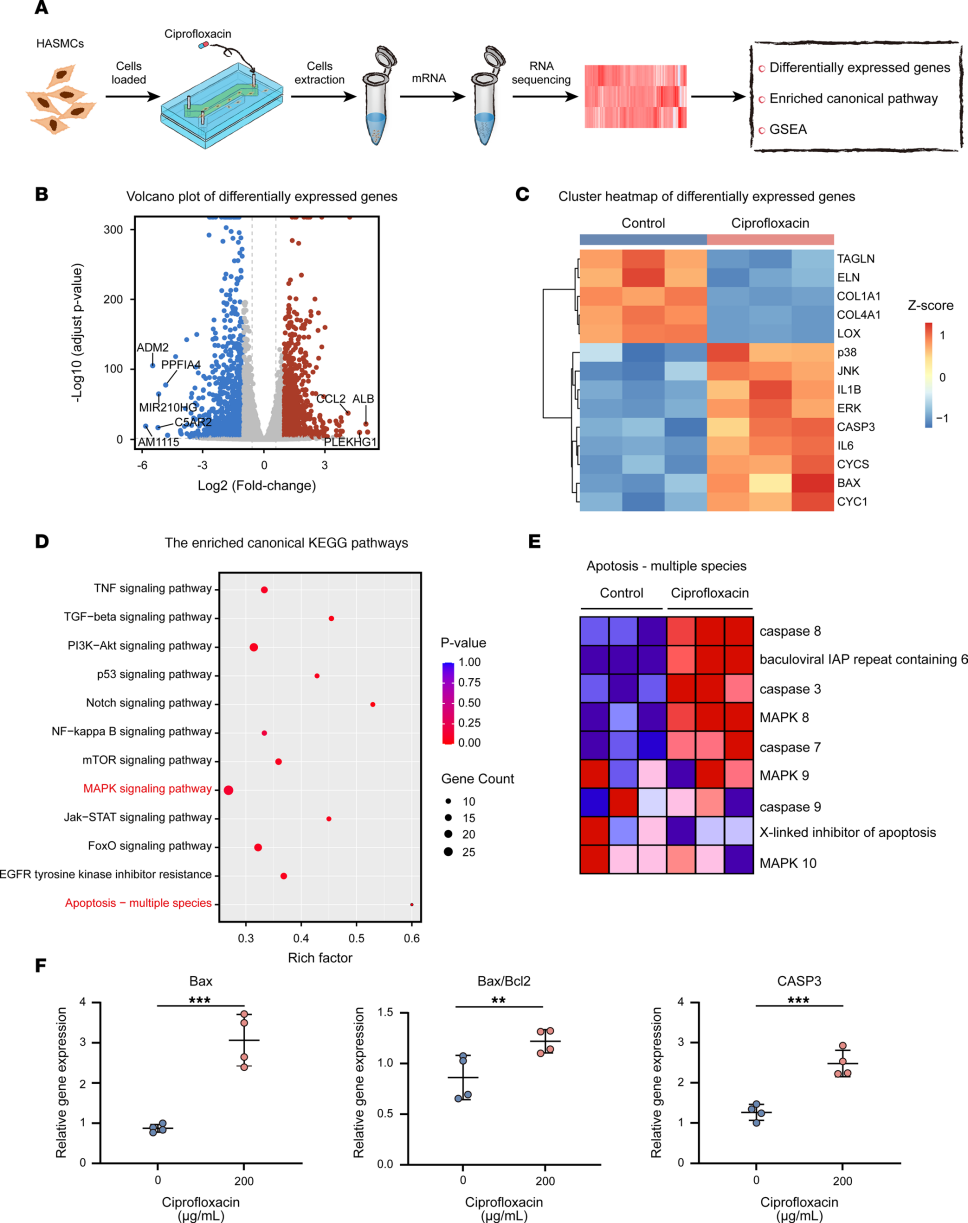
Significant apoptosis and mitochondrial dysfunction were induced in the HASMCs after ciprofloxacin treatment. (**A**) Schematic workflow of RNA-Seq analysis in the normal HASMCs under static and strain conditions in the microphysiological model after ciprofloxacin treatment at a 200 μg/mL concentration (*n* = 3). (**B**) Volcano plot showing differentially expressed genes between the ciprofloxacin and control groups. The colors indicate the following: gray, no differential expression; red, upregulated genes; and blue, downregulated genes. (**C**) Heatmap of the expression of the enriched genes involved in the contractile phenotype, degradation of the ECM, proinflammatory factors, and apoptosis as well as ERK, JNK, and P38 MAPK signaling pathways. (**D**) Enriched canonical KEGG pathways and (**E**) enriched gene set enrichment analysis identified upregulated MAPK and apoptosis-associated signaling pathways in the ciprofloxacin group compared with those in the control group. (**F**) mRNA levels of Bax, Bax/Bcl2, and caspase 3 (CASP3) measured by RT-PCR and normalized to β-actin as an internal control in the normal HASMCs (*n* = 4). **P < 0.01, ***P < 0.001 by 2-tailed Student’s *t* test.

A comparative transcriptomics analysis of the total mRNA in the normal HASMCs was performed via RNA sequencing (RNA-Seq) analysis (Figure 3A). The HASMCs were cultured under rhythmic strain conditions and divided into ciprofloxacin treatment and control groups (*n* = 3). Differential whole-gene analysis showed that ciprofloxacin treatment induced upregulation of 3573 genes and downregulation of 2226 genes (Figure 3B and Supplemental Figure 3A). Among these differentially expressed genes, significant changes were observed in the genes regulating cell phenotype, proinflammatory factors, and ECM (Figure 3C), which was consistent with the real-time polymerase chain reaction (RT-PCR) results (Supplemental Figure 2, A–C). In addition, the expression of lysyl oxidase (LOX), which regulates and stabilizes elastin, collagen, and the ECM, was significantly downregulated after ciprofloxacin treatment (Supplemental Figure 3B). The enriched canonical KEGG pathways identified via an ingenuity pathway analysis suggested that MAPK and apoptosis-associated signaling pathways were upregulated (Figure 3D). Furthermore, a gene set enrichment analysis showed that the apoptosis-associated signaling pathways were significantly upregulated, in which the MAPK signaling pathway was involved (Figure 3E and Supplemental Figure 3C). In addition, the fluorescence intensity of mitochondrial superoxide reactive oxygen species (ROS) and MitoSOX staining were upregulated in the ciprofloxacin treatment group, suggesting an increase in ROS production in the HASMCs after ciprofloxacin treatment (Supplemental Figure 4, A and B). The fluorescence intensity of mitochondrial membrane potential 5,5′,6,6′-tetrachloro-1,1′,3,3′-tetraethylbenzimi-dazolylcarbocyanine iodide (JC-1) staining of the HASMCs significantly decreased in the ciprofloxacin treatment group, indicating a decrease in the mitochondrial membrane potential in the HASMCs after ciprofloxacin treatment (Supplemental Figure 4C). In addition, the expression of apoptosis-related genes such as those encoding caspase 3 (CASP3), Bax, and cytochrome *c* (CYC) was also upregulated, which was further confirmed by RT-PCR (Figure 3F). In summary, ciprofloxacin induced mitochondrial dysfunction in the HASMCs and promoted cell apoptosis via the MAPK signaling pathway.

### Increased susceptibility of the BAV- and TAV-HASMCs to ciprofloxacin.

**Figure 4:**
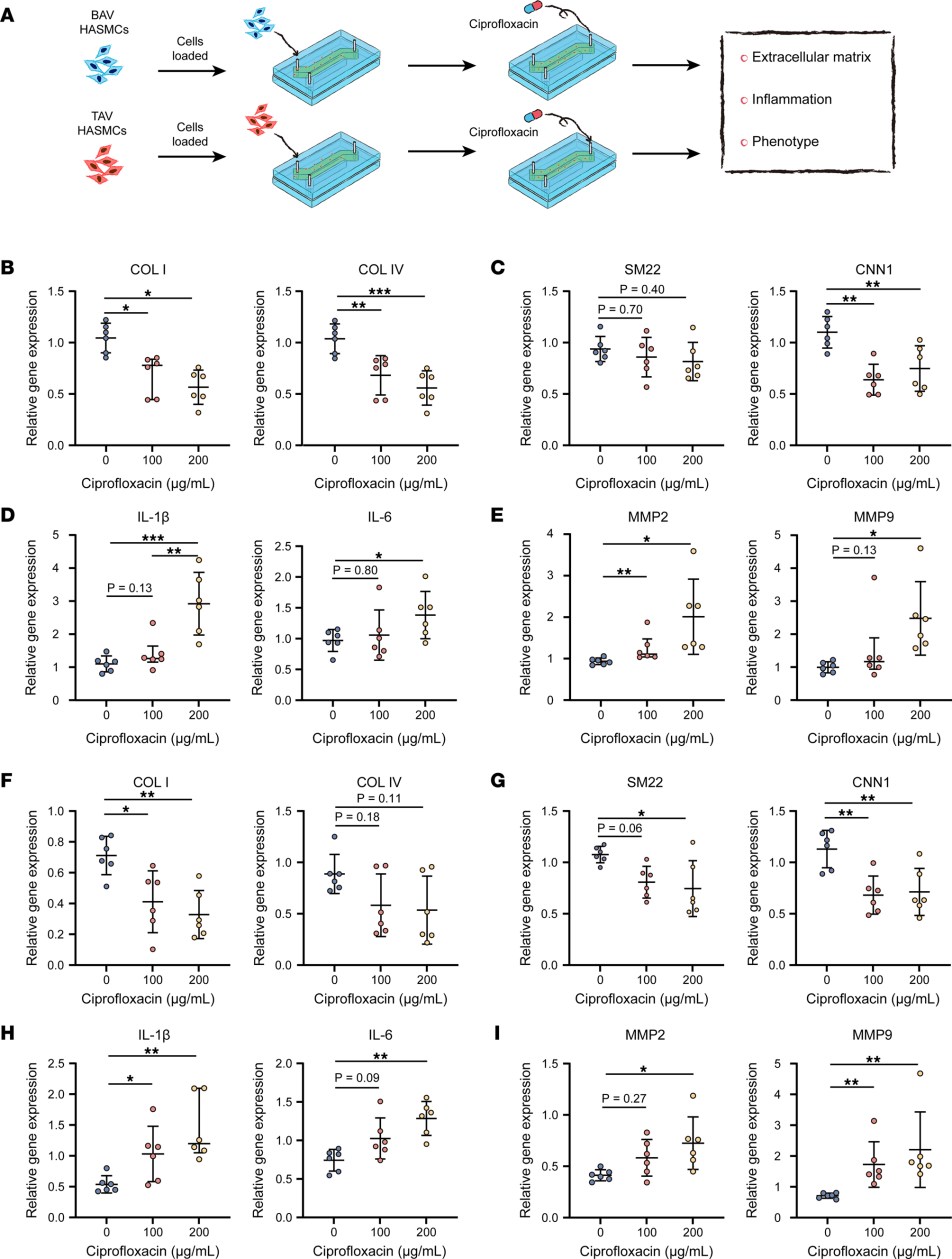
HASMCs derived from patients with BAV- and TAV-associated TAAs showed increased susceptibility to ciprofloxacin. (**A**) Schematic workflow of testing the effect of ciprofloxacin in the HASMCs derived from patients with BAV- and TAV-associated TAAs under strain conditions in the microphysiological model (*n* = 6). Relative mRNA levels of (**B**) COL I and COL IV, (**C**) SM22 and CNN1, (**D**) IL-1β and IL-6, and (**E**) MMP2 and MMP9 in the HASMCs derived from patients with BAV-associated TAA after ciprofloxacin treatment. Relative mRNA levels of (**F**) COL I and COL IV, (**G**) SM22 and CNN1, (**H**) IL-1β and IL-6, and (**I**) MMP2 and MMP9 in HASMCs derived from patients with TAV-associated TAA after ciprofloxacin treatment. *P < 0.05, **P < 0.01, ***P < 0.001 by 1-way ANOVA with Tukey’s multiple-comparison test.

The HASMCs derived from patients with BAV- and TAV-associated TAAs were isolated and cultured in the models. There were no significant differences in sex, hypertension, age, or aneurysm size between the BAV and TAV groups (Supplemental Figure 5). Alterations in the cell phenotypes, proinflammatory factors, and ECM were tested in the BAV- and TAV-associated TAA groups after ciprofloxacin treatment at different concentrations (0, 100, and 200 μg/mL) under rhythmic strain conditions (Figure 4A). In the BAV-associated TAA group, the mRNA levels of COL I, COL IV, and CNN1 were significantly downregulated at the concentration of 200 μg/mL (Figure 4, B and C); the transcription levels of IL-6, IL-1β, MMP2, and MMP9 were upregulated (Figure 4, D and E). Interestingly, the mRNA levels of COL I (*P* = 0.02), COL IV (*P* = 0.002), and CNN1 (*P* = 0.001) were significantly downregulated at the lower concentration of 100 μg/mL (Figure 4, A and B); in contrast, the mRNA level of MMP2 (*P* = 0.002) was significantly upregulated (Figure 4E). In the TAV-associated TAA group, the mRNA levels of COL I, SM22, and CNN1 decreased significantly at the concentration of 200 μg/mL (Figure 4, F and G), while the mRNA levels of IL-6, IL-1β, MMP2, and MMP9 significantly increased (Figure 4, H and I). Similarly, the transcription levels of COL I (*P* = 0.02) and CNN1 (*P* = 0.004) were significantly downregulated at the lower concentration of 100 μg/mL (Figure 4, F and G). The transcription level of IL-1β was nearly 2.0 times higher than that in the control group (*P* = 0.03, Figure 4H), while the transcription level of MMP9 was 2.5 times higher than that in the control group (*P* = 0.002, Figure 4I). Taken together, our data show that there were significant changes in the cell phenotypes, proinflammatory factors, and ECM at the lower concentration of 100 μg/mL of ciprofloxacin, whereas these changes were only found after treatment at the higher concentration of 200 μg/mL in the normal HASMCs; these findings imply that BAV- and TAV-associated TAA patient–derived HASMCs are more susceptible to ciprofloxacin.

### Effects of ciprofloxacin on the apoptosis of the HASMCs via the ERK1/2 and P38 MAPK signaling pathways.

**Figure 5:**
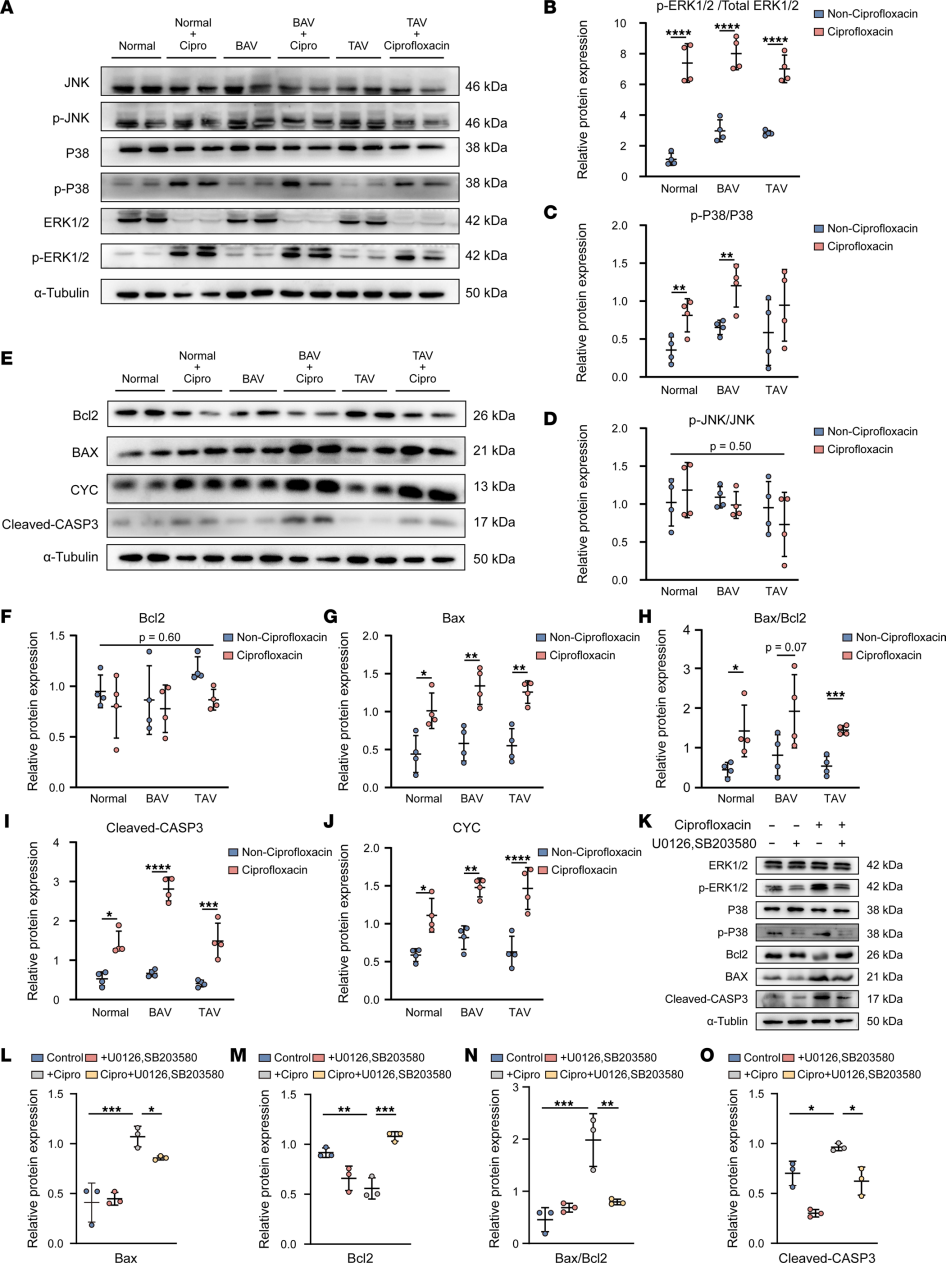
Ciprofloxacin (200 μg/mL) exacerbated the apoptosis of the HASMCs via the ERK1/2 and P38 MAPK signaling pathways. (**A**) Representative images of Western blotting of p-JNK, JNK, p-P38, P38, p-ERK1/2, and ERK1/2 and quantification of the total band densities for (**B**) p-ERK1/2/total ERK1/2, (**C**) p-P38/P38, and (**D**) p-JNK/JNK normalized to the corresponding band density of α-tubulin (*n* = 4). (**E**) Representative images of Western blotting of Bcl2, Bax, cleaved CASP3, and cytochrome *c* (CYC) and quantification of the total band densities for (**F**) Bcl2, (**G**) Bax, (**H**) Bax/Bcl2, (**I**) cleaved CASP3, and (**J**) CYC normalized to the corresponding band density of α-tubulin (*n* = 6). (**K**) Representative images of Western blotting of ERK1/2, p-ERK1/2, p-P38, P38, Bcl2, Bax, and cleaved CASP3 and quantification of the total band densities for (**L**) Bax, (**M**) Bcl2, (**N**) Bax/Bcl2, and (**O**) cleaved CASP3 normalized to the corresponding band density of α-tubulin (*n* = 3). *P < 0.05; **P < 0.01; ***P < 0.001; ****P < 0.0001 by 2-tailed Student’s *t* test (**F**–**J**) or 1-way ANOVA with Tukey’s multiple-comparison test (**L**–**O**).

To further identify the mechanism underlying ciprofloxacin-induced HASMC apoptosis, we tested the protein expression of key MAPK pathway-related proteins via Western blotting in the normal HASMCs and HASMCs derived from patients with BAV- and TAV-associated TAAs after ciprofloxacin treatment under rhythmic strain conditions. Although the phosphorylation of JNK protein, one of the proteins involved in the MAPK pathway, was not significantly altered after ciprofloxacin treatment, the phosphorylation of ERK1/2 and P38 proteins was significantly upregulated in the normal HASMCs and HASMCs derived from patients with BAV- and TAV-associated TAAs after ciprofloxacin treatment (Figure 5, A–D). The expression levels of the key apoptosis-associated proteins Bax, Bax/Bcl2, and cleaved CASP3 were upregulated in the normal and BAV- and TAV-associated TAA patient–derived HASMCs after ciprofloxacin treatment (Figure 5, E–I). Western blotting also showed that the expression level of Bax in the BAV- and TAV-associated TAA patient–derived HASMC groups was significantly higher than that in the normal group after ciprofloxacin treatment. The expression level of cleaved CASP3 was also significantly upregulated in the BAV-associated TAA patient–derived HASMC group compared with that in the normal group (Supplemental Figure 6). The expression level of CYC was significantly upregulated in all 3 groups after ciprofloxacin treatment (Figure 5J), suggesting that HASMC apoptosis may be induced by mitochondrial dysfunction–related apoptosis pathways. Furthermore, the HASMCs were also treated with P38 (SB203580) and ERK1/2 (U0126) inhibitors before ciprofloxacin treatment under rhythmic stretching. The analysis showed that the expression levels of Bax, Bax/Bcl2, and cleaved CASP3 were significantly downregulated (Figure 5, K–O), suggesting that the HASMC apoptosis induced by ciprofloxacin was regulated by the P38 and ERK1/2 MAPK pathways.

### Increased apoptosis in the aortic wall of the TAA mice after ciprofloxacin exposure.

**Figure 6:**
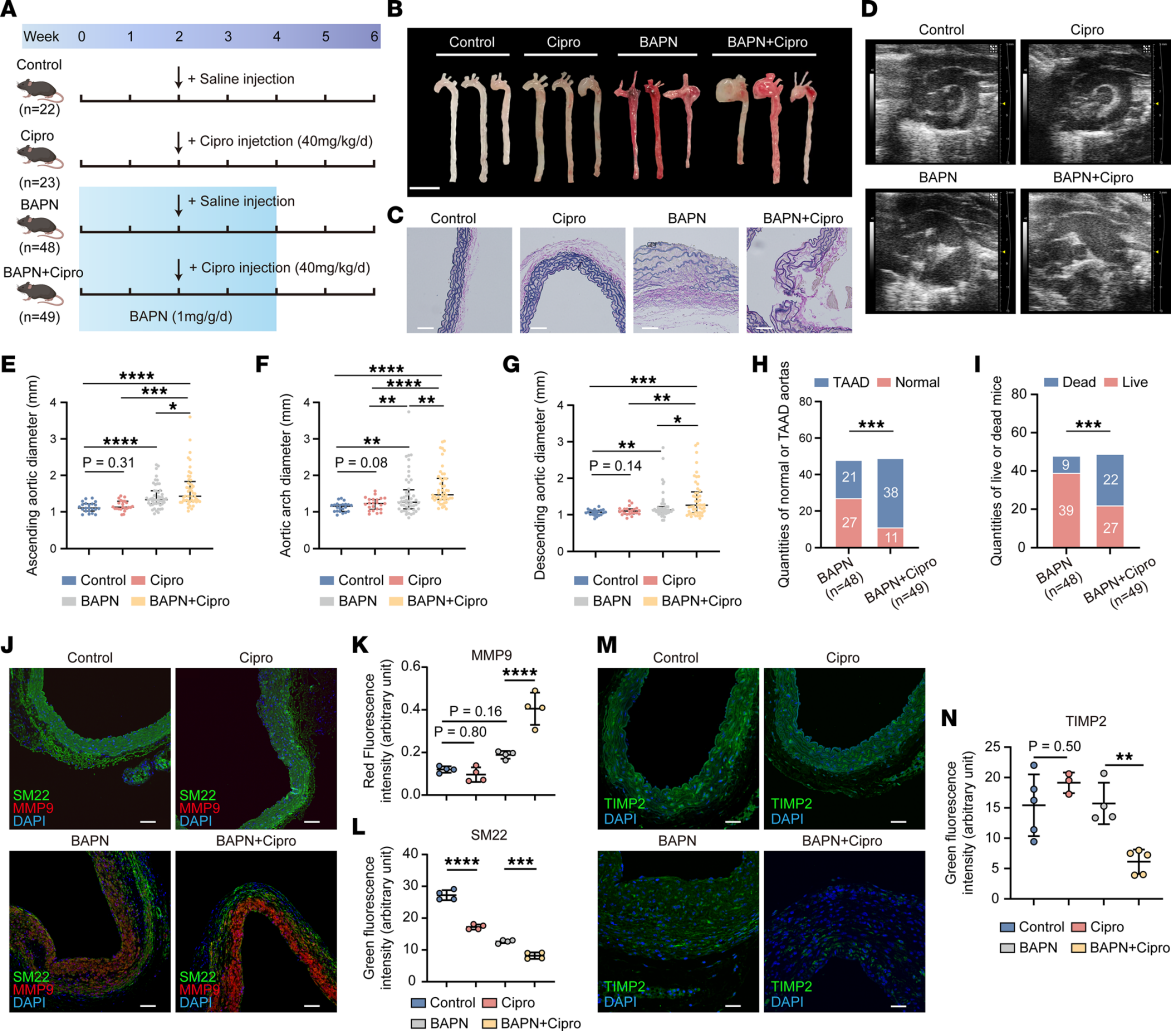
Influence of ciprofloxacin in the BAPN-induced TAA mouse models. (**A**) Schematic workflow of the animal study to verify the effect of ciprofloxacin on TAA. (**B**) Macroscopic images (scale bar: 5 mm), (**C**) elastic van Gieson–stained images (scale bars: 200 μm), and (**D**) ultrasonic images of the thoracic aortas. Statistical analysis of the diameters of (**E**) the ascending aorta, (**F**) aortic arch, and (**G**) descending aorta in the control (*n* = 22), Cipro (*n* = 23), BAPN (*n* = 48), and BAPN+Cipro (*n* = 49) groups. (**H** and **I**) Statistical analysis of the incidence of (**H**) TAAD and (**I**) death. (**J**) Immunofluorescent staining of SM22 (scale bars: 100 μm) (**K**) and MMP9 (**L**) in the thoracic aortic wall (*n* = 4). (**M** and **N**) Immunofluorescent staining of TIMP2 (scale bars: 100 μm) in the thoracic aortic wall of the control (*n* = 5), Cipro (*n* = 3), BAPN (*n* = 4), and BAPN+Cipro (*n* = 5) groups. *P < 0.05; **P < 0.01; ***P < 0.001; ****P < 0.0001 by 1-way ANOVA with Tukey’s multiple-comparison test (**E**–**G**, **K**, **L**, and **N**) or Fisher’s exact test (**H** and **I**).

**Figure 7:**
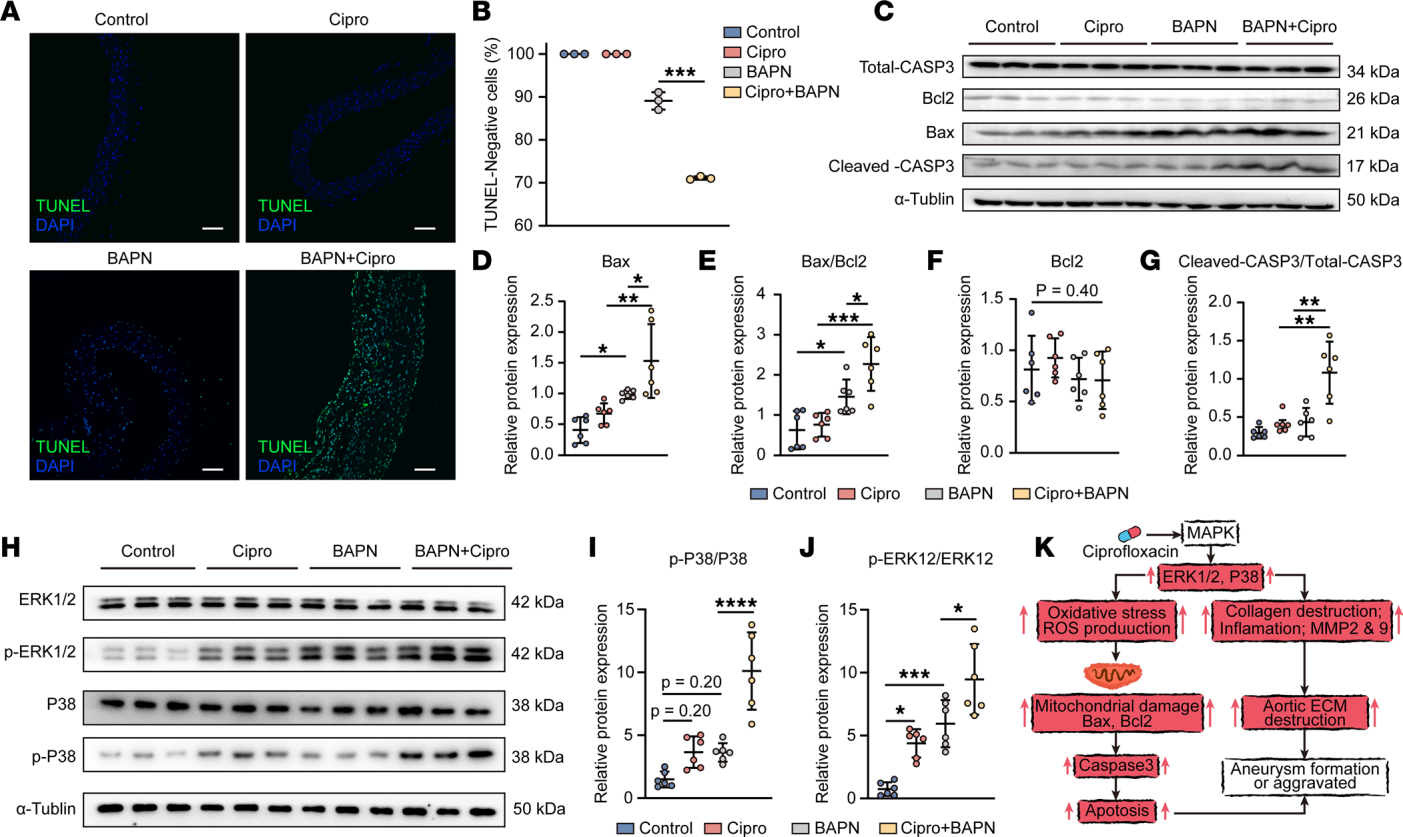
Increased apoptosis in the aortic wall of the BAPN-induced TAA mice after ciprofloxacin treatment. (**A**) Representative images of TUNEL staining in the thoracic aortic wall among the groups (*n* = 3, scale bars: 50 μm) and (**B**) quantification of TUNEL-negative cells (TUNEL-positive cells marked in green). (**C**) Representative images of Western blotting of total CASP3, Bcl2, Bax, and cleaved CASP3 and quantification of the total band densities for (**D**) Bax, (**E**) Bax/Bcl2, (**F**) Bcl2, and (**G**) cleaved CASP3/total CASP3 normalized to the corresponding band density of α-tubulin (*n* = 6). (**H**) Representative images of Western blotting of p-P38, P38, p-ERK1/2, and ERK1/2 and quantification of the total band densities for (**I**) p-P38/P38 and (**J**) p-ERK1/2/ERK1/2 normalized to the corresponding band density of α-tubulin (*n* = 6). *P < 0.05; **P < 0.01; ***P < 0.001; ****P < 0.0001 by 1-way ANOVA with Tukey’s multiple-comparison test. (**K**) Potential mechanism of ciprofloxacin-induced apoptosis and negative effects in the HASMCs of the TAA-induced mice. Ciprofloxacin could induce the degradation of COL I and COL IV, reduction in SM22 and CNN1, and upregulation of IL-1β, IL-6, MMP2, and MMP9. Meanwhile, it could induce HASMC mitochondrial dysfunction and further increase apoptosis through upregulation of Bax, Bax/Bcl2, and cleaved CASP3, presumably followed by aortic wall damage.

To partially verify the effect of ciprofloxacin on TAA progression in vivo, we constructed and treated β-aminopropionitrile–induced (BAPN-induced) TAA mouse models with ciprofloxacin. A schematic workflow of the animal experiments is shown in Figure 6A. Analysis of the gross appearance, elastic van Gieson staining, and ultrasonography of the thoracic aorta demonstrated more aggravated aortas in the BAPN+Cipro group than in the BAPN group (Figure 6, B–D). There was no significant difference in the diameter of the thoracic aorta between the control and Cipro groups; however, there were significant differences in the diameter of the ascending aorta (1.422 ± 0.33 vs. 1.632 ± 0.51 mm, *P* = 0.0275; Figure 6E), aortic arch (1.45 ± 0.57 vs. 1.674 ± 0.50 mm, *P* = 0.0012; Figure 6F), and descending aorta (1.244 ± 0.38 vs. 1.439 ± 0.52 mm, *P* = 0.038; Figure 6G) between the BAPN and BAPN+Cipro groups. The incidence of thoracic aortic aneurysm and dissection (TAAD) in the BAPN+Cipro group was significantly higher than that in the BAPN group (77.56% vs. 43.75%, *P* < 0.001; Figure 6H). In addition, the mortality in the BAPN+Cipro group was significantly higher than that in the BAPN group (44.90% vs. 23.08%, *P* < 0.001; Figure 6I). After the initial ciprofloxacin exposure, the first mouse death occurred on day 9 in the BAPN group and on day 4 in the BAPN+Cipro group, and the median survival time was 14.6 ± 3.7 and 12.7 ± 5.2 days, respectively (Supplemental Figure 7). These results indicated that deaths tended to occur earlier in the treatment group than in the control group. Thereafter, the expression level of SM22, MMP9, and TIMP2 in the aortic tissue was investigated using immunofluorescent staining. A significant increase in the expression level of MMP9 was observed in the BAPN+Cipro group compared with that in the BAPN group (Figure 6, J and K). The expression levels of SM22 and TIMP2 were significantly lower in the BAPN+Cipro group than in the BAPN group (Figure 6, J and L–N). TUNEL staining of the thoracic aorta showed that there were more TUNEL-positive cells in the BAPN+Cipro group than in the BAPN group (Figure 7, A and B). However, there was no significant difference found between the control and Cipro groups (Figure 7B). The expression levels of apoptosis-related proteins Bax, Bax/Bcl2, and cleaved CASP3 were significantly upregulated in the BAPN+Cipro group compared with those in the BAPN group (Figure 7, C–G). We further found that the phosphorylation levels of the P38 and ERK1/2 MAPK signaling pathways were upregulated in the BAPN+Cipro group compared with those in the control group (Figure 7, H–J). Taken together, our data show that ciprofloxacin induced ECM degradation and mitochondria-mediated intrinsic apoptosis of the HASMCs of the BAPN-induced TAA mice by activating the ERK1/2 and P38 MAPK pathways (Figure 7K).

### Protective effects of MAPK inhibitors on ciprofloxacin-induced aggravation of TAA in the mice.

**Figure 8:**
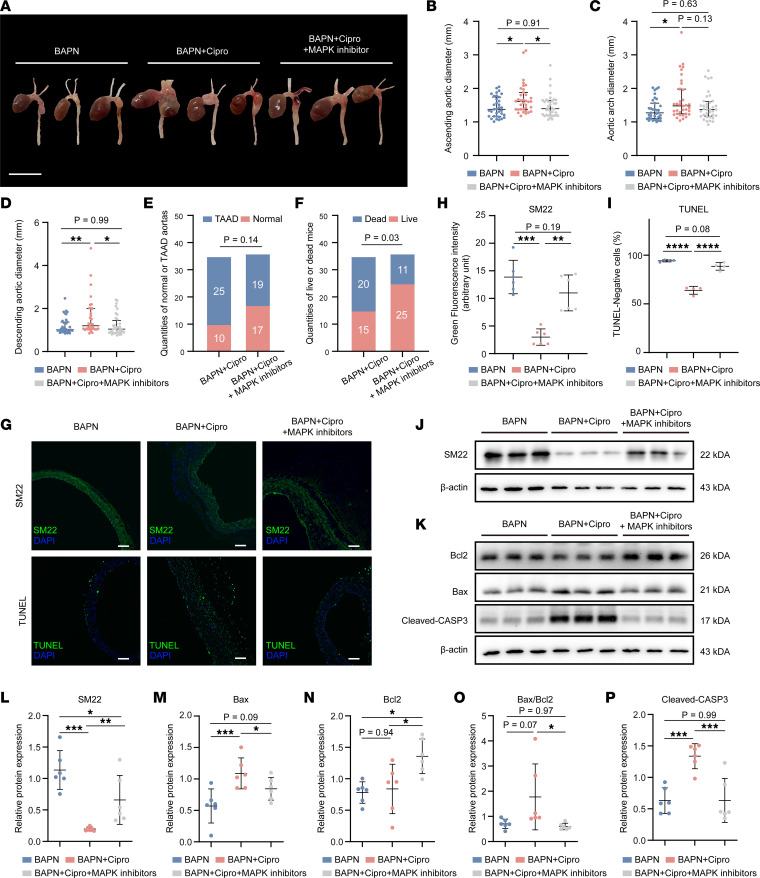
Ciprofloxacin-induced aggravation of thoracic aortic aneurysm in the mice could be alleviated by MAPK inhibitors. (**A**) Macroscopic images of the thoracic aorta. Scale bar: 5 mm. Statistical analysis of the diameters of (**B**) the ascending aorta, (**C**) aortic arch, and (**D**) descending aorta in the BAPN (*n* = 35), BAPN+Cipro (*n* = 35), and BAPN+Cipro+MAPK inhibitors (*n* = 36) groups. Statistical analysis of the incidence of (**E**) TAAD and (**F**) death between the BAPN+Cipro and BAPN+Cipro+MAPK inhibitors groups (Fisher’s exact test). (**G**) Immunofluorescent staining (scale bar: 100 μm) and (**H**) statistical analysis of SM22 in the thoracic aortic wall. (**J**) Representative images of Western blotting of SM22 and quantification of the total band densities for (**L**) SM22 normalized to the corresponding band density of β-actin (*n* = 6). (**G**) Immunofluorescent staining (scale bars: 100 μm) and (**I**) quantification of TUNEL-negative cells (TUNEL-positive cells marked in green). (**K**) Representative images of Western blotting of Bax, Bcl2, and cleaved CASP3 and total band densities for (**M**) Bax, (**N**) Bcl2, (**O**) Bax/Bcl2, and (**P**) cleaved CASP3 normalized to the corresponding band density of β-actin (*n* = 6). *P < 0.05; **P < 0.01; ***P < 0.001; ****P < 0.0001 by 1-way ANOVA with Tukey’s multiple-comparison test (**B**–**D**, **H**, **I**, and **L**–**P**).

To further validate the hypothesis that ciprofloxacin induces apoptosis through MAPK-dependent activation of Bax/Bcl2 signaling in vivo, we conducted MAPK inhibition treatment in vivo. Three-week-old C57BL/6 male mice were randomly divided into 3 groups: BAPN, BAPN+Cipro, and BAPN+Cipro+MAPK inhibitor. In all animal experiments, BAPN was administered for 28 days. Ciprofloxacin or isotonic sodium chloride solution was injected intraperitoneally from weeks 2 to 6. MAPK inhibitors (SB203580 and U0126) were administered from weeks 1 to 6 (Supplemental Figure 8). Analysis of the gross appearance of the thoracic aorta demonstrated that the aortas were healthier in the BAPN+Cipro+MAPK inhibitor group compared with that in the BAPN+Cipro group (Figure 8A). Although there was no significant difference in the diameter of the aortic arch (1.67 ± 0.61 vs. 1.46 ± 0.4 mm, *P* = 0.13) between the BAPN+Cipro and BAPN+Cipro+MAPK inhibitor groups, the diameters of the ascending aorta (1.69 ± 0.45 vs. 1.48 ± 0.36 mm, *P* = 0.04) and descending aorta (1.56 ± 0.85 vs. 1.22 ± 0.48 mm, *P* = 0.04) significantly differed (Figure 8, B–D). Although the incidence of TAAD was not significantly different between the BAPN+Cipro and BAPN+Cipro+MAPK inhibitor groups (71.43% vs. 52.78%, *P* = 0.14; Figure 8E), the mortality in the BAPN+Cipro group was significantly higher than that in the BAPN+Cipro+MAPK inhibitor group (57.14% vs. 30.56%, *P* = 0.03; Figure 8F). Immunofluorescent staining and Western blotting of the aortic wall tissues demonstrated that the expression level of SM22 significantly increased in the BAPN+Cipro+MAPK inhibitor group compared with that in the BAPN+Cipro group (Figure 8, G, H, J, and L); further, fewer TUNEL-positive cells were present in the BAPN+Cipro+MAPK inhibitor group than in the BAPN+Cipro group (Figure 8, G and I). The expression levels of Bax, Bax/Bcl2, and cleaved CASP3 were significantly downregulated in the BAPN+Cipro+MAPK inhibitor group compared with those in the BAPN+Cipro group (Figure 8, K and M–P). Therefore, the MAPK inhibitors alleviated the adverse effects of ciprofloxacin on the aortas of the BAPN-induced TAA mouse models by inhibiting apoptosis and switching the HASMC phenotype.

## Discussion

Fluoroquinolone use has been recently reported to potentially increase the risk of AA/AD. In a previous study, the comparative risk of AA/AD associated with fluoroquinolones compared to that of other antibiotics was assessed in patients who had the same types of infections with similar indication profiles (23). However, it is difficult to obtain in vivo results from patients with aortopathy who use fluoroquinolones. HASMCs are constantly subjected to hemodynamic biomechanical forces, including shear stress and cyclic strain. They are not terminally differentiated cells with high plasticity, and their cell morphology and phenotypes can be regulated by biomechanical forces, which cannot be ignored in the study of aortopathy. In this study, we fabricated an in vitro patient-derived primary HASMC–based microphysiological model to simulate the in vivo microenvironment of TAA and investigated the potential pathogenesis underlying ciprofloxacin exposure in patients with TAA.

Organ-on-a-chip models are particularly useful when it is unethical to perform studies of harmful agents in humans or when it is difficult to construct an effective animal model (24). Moreover, the species gap between human clinical studies and animal experiments cannot be ignored. The results from animal studies usually fail to predict those obtained from human clinical trials (25, 26), which hinders pharmaceutical translation in clinical practice. In our work, we used the chip model to investigate aortic diseases in relation to ciprofloxacin because this drug could potentially promote disease progression. It is unethical to conduct clinical studies to explore human responses to ciprofloxacin in patients with aortic diseases. Moreover, constructing animal models for all types of aortic diseases is difficult. For example, current animal models of BAV-associated TAA have a low penetrance. Therefore, the organ-on-a-chip model is complementary to animal models for drug screening and mechanistic studies of various aortic diseases.

BAPN-induced TAA mouse models induce fragmentation of elastic fibers and loss of smooth muscle cells (SMCs) through inhibition of LOX activity in the aortic wall (27, 28). Previous studies have reported that male mice have an increased susceptibility to AA formation compared with female mice through testosterone-mediated suppression of LOX activity (29). To increase the success rate of TAA in the mouse models, we selected male mice for model construction. Male mice have been previously used in BAPN-induced TAA models (30, 31). In clinical settings, TAA occurs more frequently in men (32). However, several large retrospective studies suggested no significant sex differences in the side effects of fluoroquinolones on AA (5, 6, 11). Thus, we used male mice for the TAA model in our study. We also used a primary HASMC–based organ-on-a-chip model and found that exposure to ciprofloxacin could induce the downregulation of LOX expression in HASMCs (Supplemental Figure 3B). This in vitro model was not treated with BAPN. Therefore, we hypothesized that ciprofloxacin could reduce the LOX activity. Further studies are needed to clarify the relationship between LOX activity and MAPK signaling in TAA animal models.

The known side effects of fluoroquinolones have been reported to be caused by ECM degradation, which is associated with stimulation of MMP activity and reduction in collagen production (33, 34). MMPs are among the best-known ECM-degrading factors and play a critical role in aortic wall remodeling (35). In addition, inflammatory cells and proinflammatory factors increase the production of MMPs, which further promotes ECM degradation and aortopathy (36). In a recent study, Guzzardi et al. used human aortic myofibroblasts exposed to ciprofloxacin and found that ciprofloxacin could induce aortic myofibroblast–mediated ECM dysregulation (37). Our results suggest that ciprofloxacin induces the upregulation of proinflammatory factors and MMP levels and reduces fibrillar collagens in normal HASMCs in a dose-dependent manner, with ciprofloxacin concentrations exceeding 200 μg/mL causing injury to normal HASMCs. However, we found that the effects of ciprofloxacin in the BAV- and TAV-associated TAA patient–derived HASMCs appeared to be more severe and sensitive than those in the normal HASMCs when treated with ciprofloxacin concentrations exceeding 100 μg/mL. In comparison, cell apoptosis was more exacerbated in the BAV- and TAV-associated TAA patient–derived HASMCs than in the normal HASMCs when treated with ciprofloxacin concentrations exceeding 200 μg/mL. Lemaire and colleagues showed that ciprofloxacin significantly increased the incidence of AD and rupture in mouse models (12, 13). In addition, ciprofloxacin decreased LOX expression and activity and increased MMP level and activity, elastic fiber fragmentation, and cell injury (12, 13). In *Fbn1*^C1041G/þ^ Marfan syndrome–induced mice, ciprofloxacin treatment accelerated aortic enlargement and increased the incidence of AD (25% vs. 47%) and rupture (5% vs. 25%) (13). Furthermore, ciprofloxacin-treated Marfan syndrome–induced mice showed higher levels of elastic fiber fragmentation, MMP expression, and apoptosis than did vehicle-treated mice (13). This study was one of the first to examine ciprofloxacin exposure in aortic diseases and lay a solid foundation for future studies. Therein, the adverse effects of ciprofloxacin treatment in animal studies on Marfan syndrome and sporadic TAA were verified. However, other types of TAA, such as BAV-associated TAA, have not been explored under ciprofloxacin treatment. Based on these studies, our work focused on identifying the effects of ciprofloxacin on primary HASMCs derived from nondiseased aortas and BAV- and TAV-associated TAA aortic tissues using an organ-on-a-chip model. Similar to the results of Lemaire et al., we also found that ciprofloxacin treatment increased the elastic fiber fragmentation, MMP expression, and apoptosis in the BAV- and TAV-associated TAA patient–derived HASMCs. Furthermore, using the microphysiological model, we found that ciprofloxacin treatment induced mitochondrial dysfunction in the HASMCs and further increased apoptosis, which was mediated by the activation of the ERK1/2 and P38 MAPK signaling pathways and could be alleviated by MAPK inhibitors in vitro and in vivo. In summary, these results indicate that ciprofloxacin induces upregulation of the expression levels of proinflammatory factors and MMPs, reduction in fibrillar collagens, and degradation of ECM in HASMCs. Accordingly, lower ciprofloxacin concentrations could induce injuries to HASMCs derived from patients with TAA. Western blotting also showed that the expression level of Bax in the HASMCs derived from patients with BAV- and TAV-associated TAA was significantly higher than that in the normal HASMCs after ciprofloxacin treatment. The expression level of cleaved CASP3 was significantly upregulated in the HASMCs derived from patients with BAV-associated TAA compared with that in the normal HASMCs. In our animal experiments, we verified that the expression levels of Bax, Bax/Bcl2, and cleaved CASP3 were significantly upregulated in the ciprofloxacin-treated TAA group compared with the ciprofloxacin-treated normal group, which is consistent with previous clinical observations (11). Thus, we hypothesized that the apoptosis in HASMCs derived from patients with BAV- and TAV-associated TAA would be more exacerbated than that in normal HASMCs.

Mitochondrial dysfunction contributes to the development of AA/AD (38–40). Herein, ciprofloxacin exposure increased mitochondrial ROS production and impaired the mitochondrial membrane potential of the HASMCs, indicating that ciprofloxacin induces mitochondrial dysfunction in HASMCs. Mitochondria play a crucial role in the regulation of apoptosis and death. The mitochondria-induced apoptosis pathway is activated by permeabilization of the outer mitochondrial membrane induced by the proapoptotic proteins Bak and Bax and promotes the release of CYC, which promotes CASP activation (41). Our study also demonstrated that ciprofloxacin exposure significantly increased the expression levels of proapoptotic proteins Bax, CYC, and CASP3, which are key proteins involved in the mitochondria-induced apoptosis pathway. These results suggest that ciprofloxacin induces mitochondrial dysfunction and promotes HASMC apoptosis via the mitochondria-related apoptosis pathway. Furthermore, the phosphorylation of ERK1/2 and P38 MAPK, which are the main components of the MAPK pathway, was enhanced after ciprofloxacin exposure, indicating that the MAPK signaling pathways were activated in HASMCs. Abnormal activation of the MAPK signaling pathways generally induces cellular oxidative stress, and senescent SMCs accumulate in the dilated ascending aorta of patients with BAV and TAV accumulation, which can activate the mitochondrial apoptosis pathways (42, 43). Therefore, ciprofloxacin could promote mitochondrial ROS production and mitochondrial dysfunction by activating the ERK1/2 and P38 MAPK pathways, ultimately resulting in HASMC apoptosis via the mitochondria-mediated intrinsic apoptosis pathway.

### Study limitations.

First, the microphysiological model of aortopathy mainly focused on the biomechanical microenvironments in the aortic wall, which is one of the key factors regulating morphological changes, differentiation, and physiological functions of HASMCs (44, 45); further, it did not utilize cocultured vascular cells, including HASMCs, aortic endothelial cells, and fibroblasts, which might better simulate the in vivo microenvironment of the human aorta. Second, no other fluoroquinolone antibiotics were used as controls. The study results can then only represent the effects of ciprofloxacin instead of other fluoroquinolones. Third, the study had a small sample size. None of the patients with TAV-associated TAA were smokers or had hyperlipidemia. Thus, future studies are needed to evaluate the effects of smoking and hyperlipidemia on cell function. Fourth, the results from the male mice used in our study may not be completely applicable to female mice. Further experiments are needed to determine whether sex has an impact on the HASMC response to fluoroquinolones. In addition, the embryological origins and properties of HASMCs in different segments of the aorta are distinct, which may result in different drug responses. The primary HASMCs used in this study were isolated from ascending aortic tissue; therefore, it remains unclear whether the pathogenesis underlying ciprofloxacin exposure is the same in other segments of the aorta.

### Conclusion.

In summary, HASMCs derived from patients with BAV- and TAV-associated TAAs were more susceptible to ciprofloxacin than normal HASMCs, which might be related to the degradation of the ECM, reduction in the contractile phenotype, and upregulation of proinflammatory factors. Ciprofloxacin exposure also induced HASMC mitochondrial dysfunction and subsequent apoptosis, presumably by activating the ERK1/2 and P38 MAPK pathways. Thus, more consideration should be given before administering ciprofloxacin to patients with BAV- and TAV-associated TAAs.

## Methods

### Isolation of primary HASMCs.

The right lateral (RL) region of the ascending aorta was selected because it is associated with a high risk of dissection or rupture, disruption of the elastic lamellae, and collapse of elastin fiber (46). Specimens from the RL region were collected from patients who underwent cardiac or ascending aortic surgery at Zhongshan Hospital, Fudan University. The specimens of 18 patients were included as follows: control nondiseased aorta (underwent coronary artery bypass grafting surgery but without aortic dilation, *n* = 6), BAV-associated TAA (*n* = 6), and TAV-associated TAA (*n* = 6). The detailed characteristics of the patients are listed in Supplemental Tables 1–3. The aortic tissues of the patients with BAV- and TAV-associated TAAs who underwent ascending aorta replacement surgery with or without aortic valve surgery were included in this study for the isolation of primary HASMCs. Patients with hereditary aortopathy, including Marfan syndrome and Loeys-Dietz syndrome, and those with traumatic aneurysm, infectious aneurysm, and AD were excluded from the study. The intima and adventitia layers of the aortic tissues were removed, and the media layer was used to harvest the HASMCs. The details of the isolation and characterization of the HASMCs are described in our previous study (20). The HASMCs were cultured in an SMC culture medium (SMCM, ScienCell) at 37°C in a 5% CO_2_ incubator. They were utilized at an early stage (P2–P6) in all experiments.

### Fabrication of the patient-derived aortic microphysiological model.

We established both BAV- and TAV-associated TAA microphysiological models based on our previous microfluidic model (20). Briefly, the model consisted of 3 layers. The top (medium channel) and bottom layers (air channel) were composed of a polydimethylsiloxane (PDMS, Sylgard 184, Dow Corning) slab containing a microchannel inside and a middle elastic PDMS membrane layer. Three layers were tightly bonded after treatment with oxygen plasma (Harrick Plasma Inc.). Subsequently, the PDMS membrane (Hangzhou Bald Advanced Materials) was coated with mouse collagen (80 mg/mL, Sigma-Aldrich). The microphysiological model was incubated for 1 hour at room temperature and dried for 2 hours at 70°C. After the collagen had dried, the model was exposed to ultraviolet radiation for 1 hour for sterilization. Before cell seeding, the medium channel was washed with phosphate-buffered saline (PBS). The HASMCs were then seeded on the PDMS membrane at a density of 2 × 10^6^/mL, cultured in the SMCM, and incubated at 37°C in a 5% CO_2_ incubator for 24 hours to allow cell attachment. Finally, the microphysiological model with HASMCs was connected to the mechanical stimulation control system, which could set the stretching frequency and amplitude. The HASMCs were stimulated by rhythmic strain with 7%–10% amplitude and 1 Hz frequency. The detailed parameters of the microphysiological model fabrication and mechanical stimulation have been previously described (20). In this study, all experiments were conducted using the microfluidic device unless otherwise stated.

### Reagent preparation.

Ciprofloxacin hydrochloride (Sigma-Aldrich), which is water soluble, was diluted in the SMCM at final concentrations of 100 and 200 μg/mL; these concentrations reflect the approximate ciprofloxacin concentrations to which the kidney is exposed and are in accordance with other study protocols of ciprofloxacin treatment for HASMCs (12, 21, 37, 47). In all experiments, the prepared 100 and 200 μg/mL ciprofloxacin solutions were treated for 24 hours, along with the start of the rhythmic strain.

### Cell viability assay.

To test the toxicity of ciprofloxacin to the normal HASMCs and determine the proper drug concentration, we cultured the cells (10,000/well) in 96-well plates (Nest). We used 0, 10, 50, 100, 150, 200, and 500 μg/mL ciprofloxacin. The viability of the HASMCs was tested using the CCK-8 kit (Dojindo) in accordance with the manufacturer’s instructions. Initially, the HASMCs were treated with or without 200 μg/mL ciprofloxacin in the microphysiological model in situ for 24 hours under rhythmic strain conditions, whereas the control cells were cultured in the microphysiological model under static conditions. Subsequently, a Live/Dead assay (Thermo Fisher Scientific) was conducted in accordance with the manufacturer’s protocol.

### Immunofluorescent staining.

Immunofluorescent staining was performed using the microphysiological model after 24 hours of rhythmic strain. Initially, the medium in the PDMS membrane channel was drawn off using a syringe, and the HASMCs were gently washed with PBS. Subsequently, the HASMCs were fixed in 4% paraformaldehyde for 30 minutes at room temperature, permeabilized with 0.2% (v/v) Triton X-100 (Beyotime) for 15 minutes, and washed 3 times with PBS. The cells were immediately blocked with bovine serum albumin (BSA; Sigma-Aldrich) for 45 minutes at room temperature. Thereafter, the cells were then incubated with a rabbit anti-TAGLN (anti-SM22) antibody (1:500 dilution; Abcam, ab14106) overnight at 4°C. The primary HASMCs were then incubated with Alexa Fluor 594–goat anti-rabbit secondary antibody (1:300 dilution; Jackson ImmunoResearch) for 1 hour at room temperature. After washing with PBS, the cells were stained with DAPI solution (1:1000 dilution; Invitrogen) for 10 minutes and washed with PBS. In addition, F-actin (rhodamine phalloidin; Thermo Fisher Scientific, R415), a directly labeled antibody, was diluted in 1% BSA to obtain a 0.2 U/mL concentration of F-actin solution. After permeabilization with Triton X-100, the HASMCs were incubated in 0.2 U/mL F-actin solution for 1 hour at room temperature, followed by PBS washing and DAPI staining as described above. Images were acquired using a fluorescence microscope (Olympus). All images were analyzed using ImageJ software (NIH).

### Membrane potential, ROS, and MitoSOX analyses of the mitochondria.

To assess the function of the mitochondria, we used 3 types of fluorescent dyes: mitochondrial membrane potential assay kit (JC-1, a cyanine dye used for mitochondrial membrane potential testing; Beyotime), Fluorometric Intracellular ROS Kit (ROS, Sigma-Aldrich), and MitoSOX (Thermo Fisher Scientific). Before staining with these fluorescent dyes, the cells were treated with ciprofloxacin for 24 hours under rhythmic strain conditions in the microphysiological model. The tests were performed in accordance with the manufacturer’s guidelines. After incubation with the fluorescent dye, the HASMCs were washed with PBS, followed by nuclear staining with Hoechst (1:1000 dilution, Thermo Fisher Scientific) at 37°C in a 5% CO_2_ incubator for 5 minutes. All staining images were captured using a confocal laser microscope (Olympus) and analyzed using ImageJ software.

### Quantitative RT-PCR.

Total RNA was extracted from the HASMCs using an RNA-Quick Purification Kit (Escience) in accordance with the manufacturer’s protocol after ciprofloxacin treatment for 24 hours under rhythmic strain conditions. Five hundred nanograms of total RNA was reverse transcribed into cDNA using PrimeScript RT Master Mix (Takara Biomedical Technology). One microliter of diluted cDNA was loaded along with Hieff UNICON qPCR SYBR Green Master Mix (YEASEN) and gene-specific primers (Shanghai Huagene Bio-Technology) in a 96-well plate for subsequent RT-PCR analysis, following the manufacturer’s instructions. The details of the specific gene primers are listed in Supplemental Table 4.

### RNA-Seq.

Three normal HASMC lines with or without ciprofloxacin treatment (200 μg/mL) were cultured in the microphysiological model under rhythmic strain and underwent RNA-Seq. Initially, the total RNA was purified using TRIzol reagent (Invitrogen) in accordance with the manufacturer’s protocol. RNA purity was confirmed using Dynabeads Oligo (dT) (Thermo Fisher Scientific, 25-61005) using 2 rounds of purification. RNA integrity was assessed using Bioanalyzer 2100 (Agilent Technologies). Briefly, the mRNA was purified, fragmented, and used for first- and second-strand cDNA syntheses. The average insert size of the final cDNA library was 300 ± 50 base pairs (bp). Finally, we performed 2 × 150-bp paired-end sequencing (PE150) on an Illumina NovaSeq 6000 (LC Sciences) in accordance with the manufacturer’s instructions. The differentially expressed mRNAs were selected with a fold change of greater than 2 or less than 0.5 and with a parametric *F* test comparing the nested linear models (*P* < 0.05) using the R package edgeR (https://bioconductor.org/packages/release/bioc/html/edgeR.html). The RNA-Seq data, including raw data, have been deposited to the National Genomics Data Center, China National Center for Bioinformation/Beijing Institute of Genomics (https://ngdc.cncb.ac.cn/gsa-human/s/11R70rR9) with the data set Bioproject number PRJCA011559.

### Western blotting.

The cells were lysed using RIPA lysis buffer (Beyotime), together with phenylmethylsulfonyl fluoride (Beyotime) and phosphatase inhibitors (Thermo Fisher Scientific). Cell extracts were separated by 12% SDS-PAGE (Beyotime) and transferred to polyvinylidene difluoride (PVDF) membranes (MilliporeSigma) in accordance with the manufacturer’s instructions. After transfer, the PVDF membranes were blocked with 5% skim milk in TBST and subsequently incubated with primary antibodies (detailed primary antibody information shown in Supplemental Table 5) overnight at 4°C. The sections were then incubated with goat anti-mouse or goat anti-rabbit IgG secondary antibodies (Jackson ImmunoResearch) at a 1:5000 dilution in 5% skim milk/TBST for 1 hour at room temperature. The bands were detected using the SuperSignal chemiluminescence reagent substrate (Tanon Femto-sig ECL, ABclonal Technology). α-Tubulin or β-actin was used as the normalizing housekeeping protein. Band intensity was quantified using ImageJ software.

### Animal study.

The 3-week-old C57BL/6 male mice were randomly divided into 4 groups: control group (*n* = 22, routine dietary water with intraperitoneal injection of isotonic sodium chloride solution), Cipro group (*n* = 23, routine dietary water with intraperitoneal injection of ciprofloxacin), BAPN (Aladdin) (1 mg/g/d) group (*n* = 48, BAPN dissolved in drinking water with intraperitoneal injection of isotonic sodium chloride solution), and BAPN+Cipro group (*n* = 49, BAPN dissolved in drinking water with intraperitoneal injection of ciprofloxacin). We selected the concentration of ciprofloxacin according to the standard low dose (8.3 mg/kg/d) for the treatment of patients with common indications and converted it to the equivalent dose concentration in mice (40 mg/kg/d, intraperitoneal injection) (48–50). In all animal experiments, BAPN diluted in daily drinking water was used for 28 days. Ciprofloxacin or isotonic sodium chloride solution was injected intraperitoneally from weeks 2 to 6. AA/AD was defined as previously described (12). At the end of the study, the mice were sacrificed, and the aortas were processed for AA/AD evaluation and tissue analysis.

We validated the hypothesis that ciprofloxacin could induce apoptosis through MAPK-dependent activation of Bax/Bcl2 signaling in vivo. Three-week-old C57BL/6 male mice were randomly divided into 3 groups: the BAPN group (*n* = 35, BAPN dissolved in drinking water with intraperitoneal injection of isotonic sodium chloride solution), BAPN+Cipro group (*n* = 35, BAPN dissolved in drinking water with intraperitoneal injection of ciprofloxacin), and BAPN+Cipro+MAPK inhibitor group (*n* = 36, BAPN dissolved in drinking water with intraperitoneal injection of ciprofloxacin and SB203580 [0.5 mg/kg/d] and U0126 [10 mg/kg/d]). In all animal experiments, BAPN was administered for 28 days. Ciprofloxacin or isotonic sodium chloride solution was injected intraperitoneally from weeks 2 to 6, while inhibitors were administered from weeks 1 to 6.

### Statistics.

Categoric data were compared between groups by using the χ^2^ test or Fisher’s exact test. Continuous data were evaluated for distribution normality by using the Shapiro-Wilk test. Any data that were normally distributed were summarized as median ± standard deviation (SD) and compared using a Student’s *t* test or 1-way ANOVA with Tukey’s multiple-comparison test. Any data that were in violation of the normality assumption were summarized as median and interquartile range and evaluated using a Mann-Whitney test. A *P* value of less than 0.05 was considered statistically significant: *P < 0.05, **P < 0.01, ***P < 0.001, ****P < 0.0001. Statistical analyses were conducted using Prism 8.0 (GraphPad Software).

### Study approval.

Human aortic specimens were utilized under approvals of Zhongshan Hospital, Fudan University Ethics Committee (no. B2020-158). Written informed consent was obtained from all patients before participation. Animal studies were conducted under approvals of Zhongshan Hospital, Fudan University Ethics Committee (no. 2021-043).

## Author contributions

BX, MA, and KZ conceived the research. Patient enrolment, clinical phenotype data collation, sample acquisition preparation, and intellectual contribution was performed by all authors. The manuscript was written BX and MA, and revised by KZ, JL, and CW. All authors critically reviewed the manuscript and approved the final submitted version.

## Supplementary Material

Supplemental data

## Version 1 12/06/2022

In-Press Preview

## Version 2 01/24/2023

Electronic publication
