# Supplementary material for: Ciprofloxacin exacerbates dysfunction of smooth muscle cells in a microphysiological model of thoracic aortic aneurysm
Source: JCI Insight. 2023 Jan 24;8(2):e161729. doi: 10.1172/jci.insight.161729 (PMC9977303; doi:10.1172/jci.insight.161729)
Supplement: Supplemental data [file jciinsight-8-161729-s009.pdf]

Supplementary Figures, Figure legends and Tables

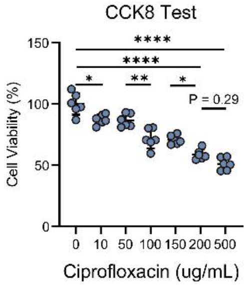

Supplementary Figure 1. Effect of ciprofloxacin on normal HASMC viability. The normal HASMC viability was tested using CCK-8 assay at concentrations of 0, 10, 50, 100, 150, 200, and 500 µg/mL of ciprofloxacin (n = 6). ($^{*}P < 0.05$, $^{**}P < 0.01$, $^{****}P < 0.0001$, 1-way ANOVA)

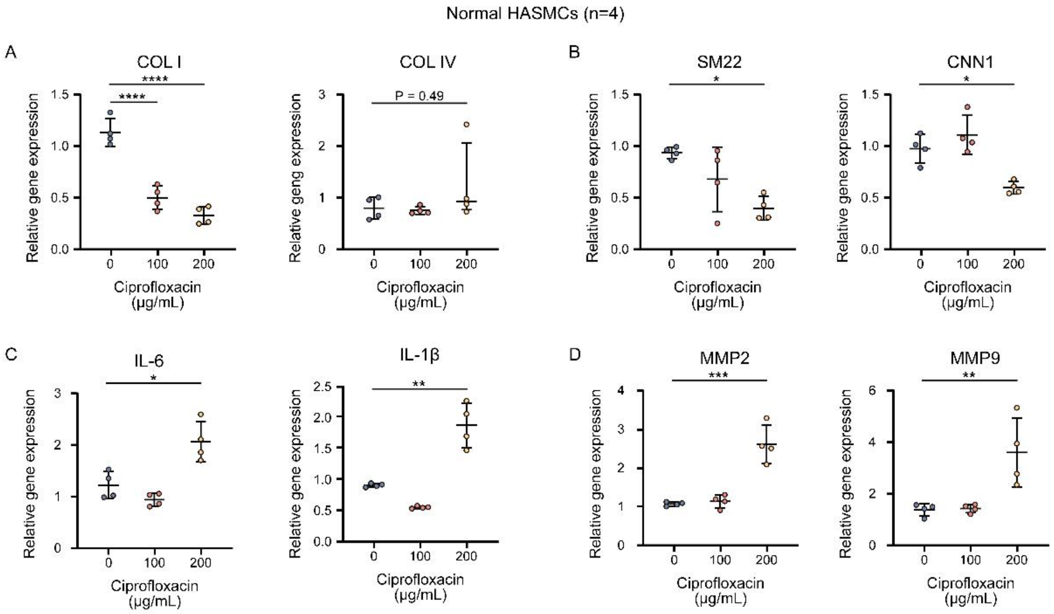

Supplementary Figure 2. Representative images of the mRNA levels of (A) COL I and COL IV, (B) SM22 and CNN1, (C) IL-6 and IL-1β, and (D) MMP2 and MMP9 measured by RT-PCR and normalized to β-actin as an internal control in the normal HASMCs (n = 4). ($^{*}P < 0.05$, $^{**}P < 0.01$, $^{****}P < 0.0001$, 1-way ANOVA)

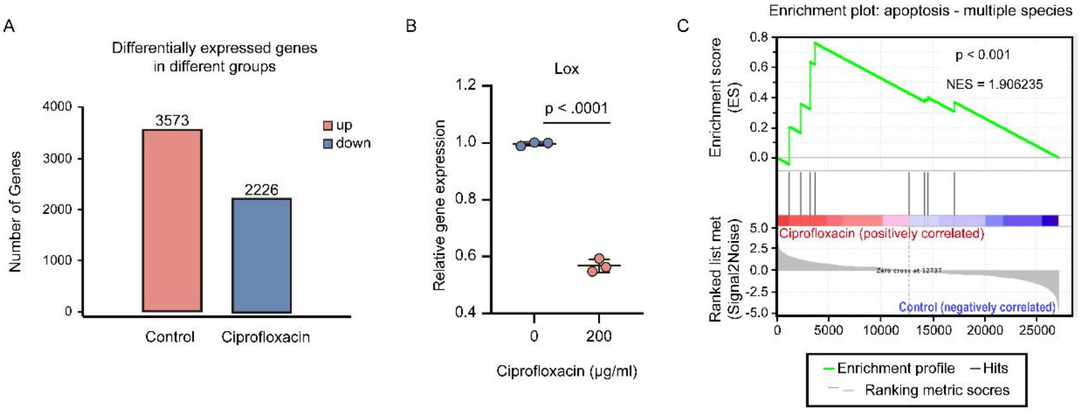

Supplementary Figure 3. RNA-seq analysis in the normal HASMCs under static and strain conditions in the microphysiological model after ciprofloxacin treatment (n = 3). (A) Differentially expressed gene analysis showed that 3573 genes were upregulated, and 2226 genes were downregulated in the ciprofloxacin group compared with those in the control group. (B) Expression level of lysyl oxidase (LOX) on RNA-seq. (C) Enriched gene set enrichment analysis identified upregulated apoptosis-associated signaling pathways in the ciprofloxacin group compared with those in the control group.

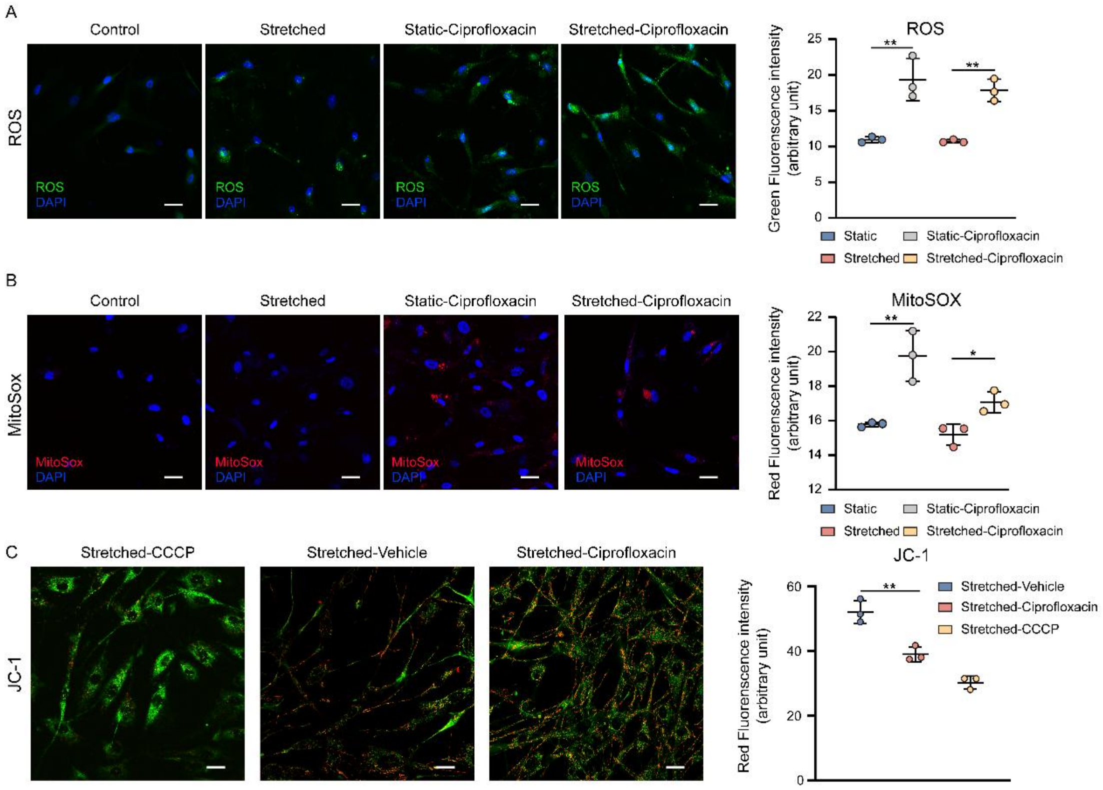

Supplementary Figure 4. Ciprofloxacin treatment induced mitochondrial dysfunction in the

HASMCs (n = 3). (A) Reactive oxygen species (ROS) staining of the mitochondria in the normal HASMCs in the ciprofloxacin and control groups under strain and static conditions and quantification of the relative ROS fluorescence intensity. Scale bar: 20 µm. (B) MitoSOX staining of mitochondrial superoxide generation in the normal HASMCs in the ciprofloxacin and control groups under strain and static conditions and quantification of the relative MitoSOX fluorescence intensity. Scale bar: 20 µm. (C) JC-1 staining of the mitochondrial membrane potentials in the normal HASMCs in the ciprofloxacin and control groups under strain conditions and relative red fluorescence intensity. Scale bar: 20 µm. CCCP indicates a positive control, and vehicle indicates a negative control. ($^{*}P < 0.05$, $^{**}P < 0.01$, Student's t test, two-tailed)

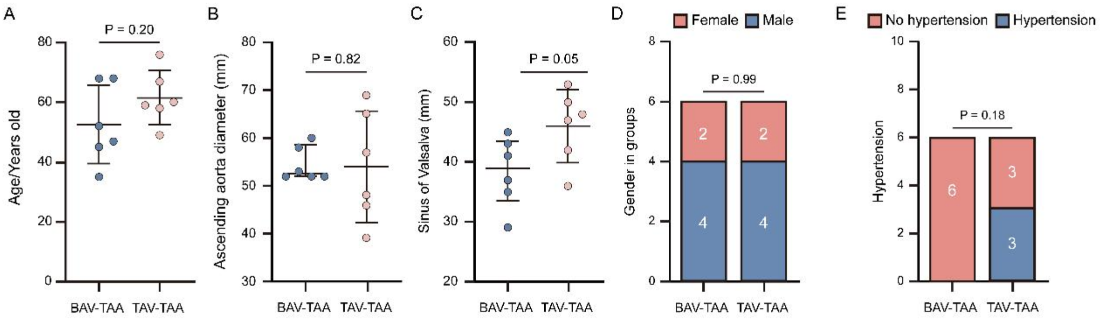

Supplementary Figure 5. Comparison of baseline data between the BAV- and TAV-associated TAA groups (n = 6). There was no significant difference in (A) age, (B) diameter of the ascending aorta and (C) sinus of valsalva, (D) sex, and (E) hypertension. Categorical data were compared between groups using Fisher's exact test. Continuous data were evaluated for distribution normality using the Shapiro–Wilk test. Normally distributed data were compared using a t test. Non-normally distributed data were evaluated using the Mann–Whitney test.

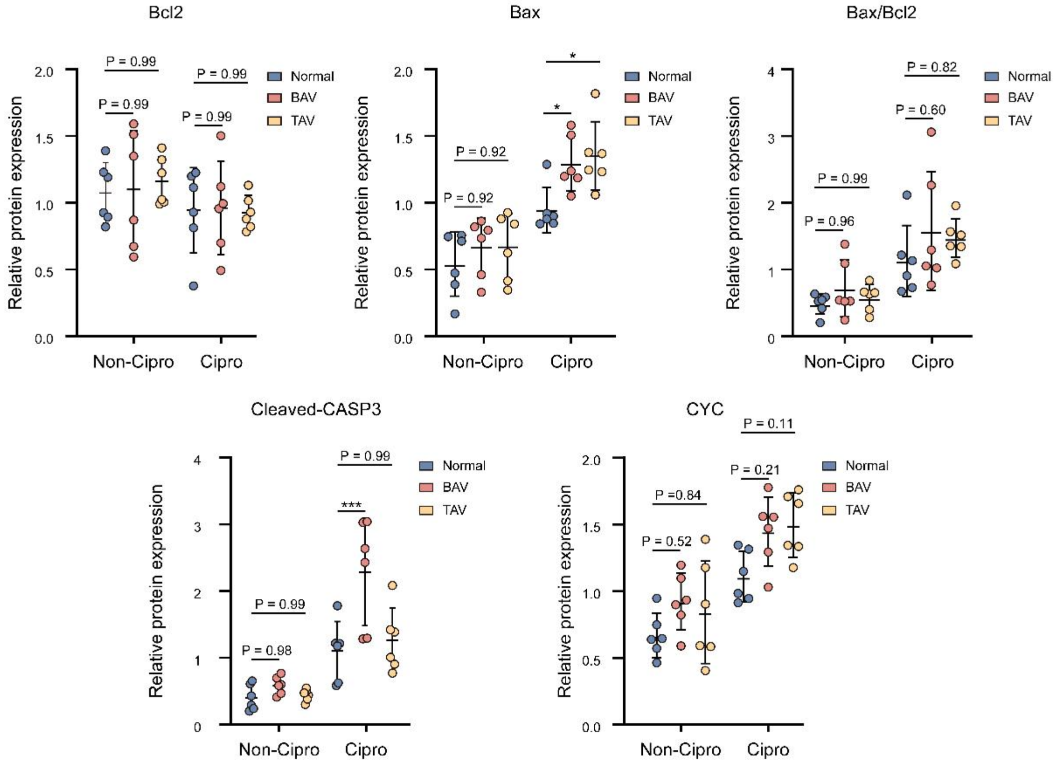

Supplementary Figure 6. Reanalysis of the western blotting results (Figure 5E) of the differences between the ciprofloxacin-treated healthy cells and ciprofloxacin-treated diseased cells (n = 6). Representative images of western blotting of Bcl2, Bax, cleaved CASP3, and cytochrome C (CYC) and quantification of the total band densities for Bcl2, Bax, Bax/Bcl2, cleaved CASP3, and CYC normalized to the corresponding band density of α-tubulin. ($^{*}P < 0.05$, $^{**}P < 0.01$, $^{***}P < 0.001$, $^{****}P < 0.0001$, 1-way ANOVA)

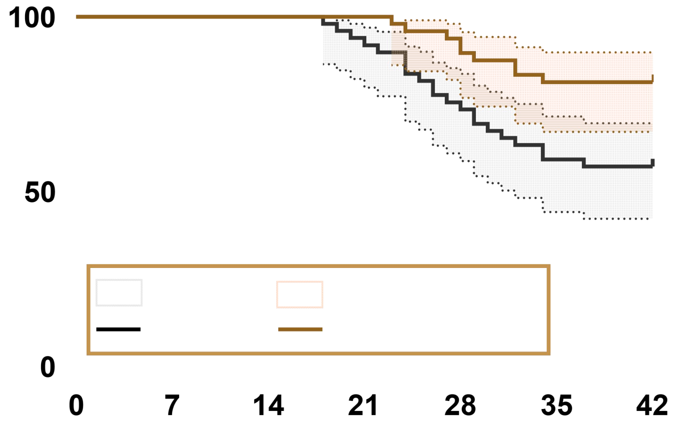

Supplementary Figure 7. Survival curves for the mouse experiments between the BAPN and BAPN+Cipro groups. Comparison of the Kaplan–Meier survival curves between the BAPN and BAPN+Cipro groups treated with ciprofloxacin or the vehicle. (Chi square test). HR, hazard ratio. CI, confidence interval.

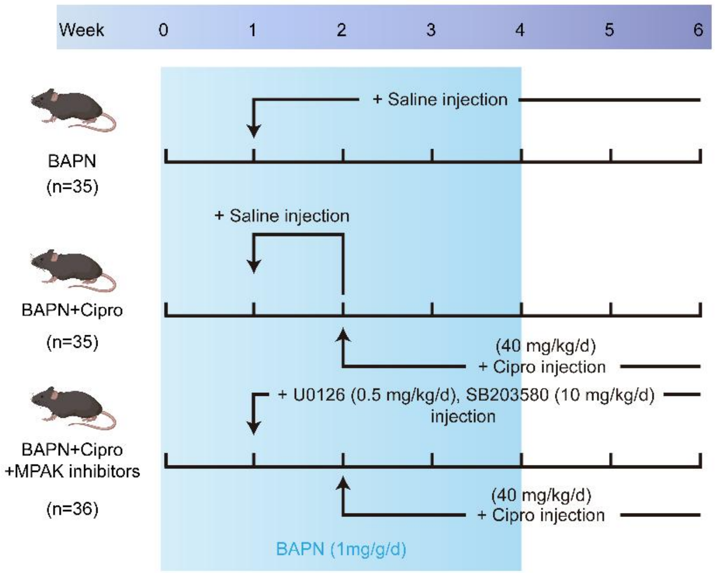

Supplementary Figure 8. Schematic workflow of the animal study to verify the hypothesis that ciprofloxacin induces apoptosis through MAPK-dependent activation of Bax/Bcl2 signaling.

Supplementary table 1. Characteristics of the patient cohort with non-diseased aortas.

Variables	N1	N2	N3	N4	N5	N6
**Age (y)**	61	70	62	51	55	74
**Gender**	Male	Male	Female	Female	Male	Male
**Hypertension**	+	+	-	-	+	+
**Diabetes**	-	-	+	-	-	-
**Hyperlipidemia**	-	-	-	-	-	-
**Aortic valve function**						
**AS: None/Mild/Moderate-Severe/Severe**	N	N	N	N	N	N
**AR: None/Mild/Moderate-Severe/Severe**	N	N	N	N	N	N
**Ascending aorta diameter (mm)**	32	29	30	28	33	28
**Sinus of Valsalva (mm)**	36	32	36	31	39	29
**Operation methods**	CABG	CABG	CABG	CABG	CABG	CABG

N1, N2, N3, N4, N5, N6, Patient #1, #2, #3, #4, #5, #6 in non-diseased group. AS, aortic stenosis; AR, aortic regurgitation; N, None; M, Mild; M-S, Moderate-Severe, S, Severe; CABG, Coronary Artery Bypass Graft.

Supplementary table 2. Characteristics of the patient cohort with BAV-TAA patients.

Variables	B1	B2	B3	B4	B5	B6
**Age (y)**	47	52	68	68	45	35
**Gender**	Female	Female	Male	Male	Male	Male
**Hypertension**	-	-	-	-	-	-
**Diabetes**	-	-	-	-	-	-
**Smoking**	-	-	-	-	-	-
**Hyperlipidemia**	-	-	-	-	-	-
**Left ventricular ejection fraction**	59	66	58	65	71	58
**Aortic valve function**						
**AS: None/Mild/Moderate-Severe/Severe**	N	N	N	M	N	N
**AR: None/Mild/Moderate-Severe/Severe**	N	M	M-S	M	M	M
**Ascending aorta diameter (mm)**	52	58	53	52	60	52
**Sinus of Valsalva (mm)**	29	37	43	35	45	41
**Operation methods**	AAR	AAR	Bentall	Wheat's	David	AAR
**Concomitant valve surgery**	AVP	-	AVR	AVR	-	-
**Concomitant CABG**	-	-	-	-	-	-

BAV, bicuspid aortic valve; TAV, tricuspid aortic valve; B1, B2, B3, B4, B5, B6, BAV- TAA Patient #1,

#2, #3, #4, #5, #6. AS, aortic stenosis; AR, aortic regurgitation; N, None; M, Mild; M-S, Moderate-Severe; AVR, Aortic Valve Replacement; AVP, Aortic Valvular Valvuloplasty. S, Severe; AA, AAR Ascending Aorta Replacement. CABG, Coronary Artery Bypass Graft.

Supplementary table 3. Characteristics of the patient cohort with TAV-TAA patients.

Variables	T1	T2	T3	T4	T5	T6
**Age (y)**	49	59	76	58	60	67
**Gender**	Male	Male	Male	Male	Female	Female
**Hypertension**	-	+	+	-	-	+
**Diabetes**	-	-	-	-	-	-
**Smoking**	-	-	-	-	-	-
**Hyperlipidemia**	-	-	-	-	-	-
**Left ventricular ejection fraction**	62	65	62	63	54	65
**Aortic valve function**						
**AS: None/Mild/Moderate-Severe/Severe**	N	N	N	N	N	N
**AR: None/Mild/Moderate-Severe/Severe**	M-S	S	M	S	S	M
**Ascending aorta diameter (mm)**	46	48	57	39	65	69
**Sinus of Valsalva (mm)**	53	48	42	47	50	36
**Operation methods**	David	Bentall	AAR	David	Bentall	Wheat’s
**Concomitant valve surgery**	-	AVR	-	-	AVR	AVR
**Concomitant CABG**	-	-	-	-	-	-

T1, T2, T3, T4, T5, T6, TAV-TAA Patient #1, #2, #3, #4, #5, #6.

Supplementary table 4. Primer sequences used for RT-PCR.

Primer Name	Forward (5' to 3')	Reverse (5' to 3')
COL I	GAGGGCCAAGACGAAGACATC	CAGATCACGTCATCGCACAAC
COL IV	GGGATGCTGTTGAAAGGTGAA	GGTGGTCCGGTAAATCCTGG
IL-1β	AGCTACGAATCTCCGACCAC	CGTTATCCCATGTGTCGAAGAA
IL6	ACTCACCTCTTCAGAACGAATTG	CCATCTTTGGAAGGTTCAGGTTG
SM22	CCGTGGAGATCCCAACTGG	CCATCTGAAGGCCAATGACAT
CNN1	CTCCATTGACTCGAACGACTC	CAGGTCTGCGAAACTTCTTAGA
MMP2	TACAGGATCATTGGCTACACACC	GGTCACATCGCTCCAGACT
MMP9	AGACCTGGGCAGATTCCAAAC	CGGCAAGTCTTCCGAGTAGT
B-actin	CATGTACGTTGCTATCCAGGC	CTCCTTAATGTCACGCACGAT
Casepase 3	CATGGAAGCGAATCAATGGACT	CTGTACCAGACCGAGATGTCA
Bax	CCCGAGAGGTCTTTTTCCGAG	CCAGCCCATGATGGTTCTGAT
Bcl2	GGTGGGGTCATGTGTGTGG	CGGTTCAGGTACTCAGTCATCC

Supplementary table 5. Antibodies used for Western blot.

Antibody name	Cat no	Company
α-Tublin	AF0001	Cell Signaling TECHNOLOGY
JNK	CY5490	Abways Technology
p-JNK	CY5541	Abways Technology
P38	8690S	Cell Signaling TECHNOLOGY
p-P38	4511S	Cell Signaling TECHNOLOGY
ERK1/2	AB3373	Abways Technology
p- ERK1/2	28733-1-AP	Proteintech
Cleaved-Caspase 3	9964S	Cell Signaling TECHNOLOGY
Bax	60267-1-Ig	Proteintech
Bcl2	Ab32124	Abcam
CYC	66264-1-Ig	Proteintech
TIMP1	CY3822	Abways Technology
TIMP2	CY3823	Abways Technology
IL-1β	12242	Cell Signaling TECHNOLOGY
CNN1	Ab46794	Abcam
SM22	Ab14106	Abcam
MMP9	Ab58803	Abcam
B-actin	8457S	Cell Signaling TECHNOLOGY
